# Independent Assessment of Point of Care Ultrasound (POCUS) Training Among Graduating Residents in Two Anesthesiology Residency Programs

**DOI:** 10.7759/cureus.97339

**Published:** 2025-11-20

**Authors:** Sudhakar Subramani, Richard Gardner, Alexander Kaizer, Joss J Thomas, Sudarshan Setty

**Affiliations:** 1 Anesthesiology, University of California, Irvine, USA; 2 Anesthesiology &amp; Perioperative Medicine, University Hospitals Cleveland Medical Center, Cleveland, USA; 3 Biostatistic and Informatics, Colorado School of Public Health, University of Colorado-Anschutz Medical Campus, Aurora, USA; 4 Anesthesiology, University of Minnesota, Minneapolis, USA

**Keywords:** external assessment, pocus, pocus curriculum, resident education, unbiased evaluation

## Abstract

Background

In the recent past, point-of-care ultrasound (POCUS) has been an integral part of resident education in anesthesiology. Residency programs have varied levels of POCUS curriculum. To assess the effectiveness of POCUS training, we conducted a study on graduating residents of two academic anesthesiology programs. To avoid bias, we used an external examiner to assess the effectiveness of teaching.

Methods

Graduating anesthesiology residents from the University of Minnesota (12 residents ) and the University of Iowa (24 residents) from 2022 and 2023 were included. Our study collected data on the different aspects of POCUS training: knowledge, ability to acquire and interpret images, and management of patients based on POCUS imaging in four different stations. The stations included multiple-choice questions (MCQs), image interpretation and patient management, image acquisition, and post-test surveys.

Results

There was no statistically significant difference in the knowledge, image interpretation, and post-test surveys between the two programs. Residents from the University of Minnesota performed slightly better in some areas than their University of Iowa counterparts in acquiring images in subcostal, parasternal short, parasternal long, and pneumothorax assessments.

Conclusions

Differences in teaching curriculum and model body habitus may account for the observed differences between the two groups. A structured POCUS curriculum with an unbiased means of assessing the effectiveness of teaching can be valuable in improving teaching methods and providing feedback to the learners. A study with additional cohorts, more sites, or a larger number of participants may have greater statistical power to detect potential differences between sites or cohorts.

## Introduction

Point-of-care ultrasound (POCUS) represents a significant advancement in medical diagnostics, allowing clinicians to rapidly obtain reliable and accurate bedside assessments of patients. This technology has been widely adopted across numerous specialties, including anesthesia, where its applications have become integral to daily practice [1]. The versatility of POCUS enables anesthesia providers to navigate cardiovascular anatomy safely and efficiently, aiding in the identification of common cardiac conditions that can lead to perioperative hemodynamic instability. Beyond cardiac assessments, POCUS has expanded to include lung, gastric, and Focused Assessment with Sonography for Trauma (FAST) examinations, facilitating the identification of certain intra-abdominal structures in acute care management as well as for determining gastric content prior to anesthetic induction in special circumstances [2].

Recognizing the critical role of POCUS in modern medical practice, the Accreditation Council for Graduate Medical Education (ACGME) has integrated POCUS as a core competency and milestone within the anesthesiology residency training curriculum [3]. Furthermore, POCUS has been incorporated into the Objective Structured Clinical Examinations (OSCE) portion of the APPLIED exam administered by the American Board of Anesthesiology (ABA) since 2022 [4]. This inclusion underscores the importance of POCUS proficiency for graduating anesthesiology residents, ensuring they are equipped with the necessary skills to utilize this technology effectively in clinical settings.

To meet the growing demand for POCUS proficiency, medical schools and residency programs have begun incorporating structured POCUS training into their curricula [5-7]. Despite the widespread adoption of POCUS training, there remains a lack of standardized methodologies for teaching and assessing competency. The OSCE has emerged as a key tool for evaluating learners' manual and interpersonal skills in a controlled and standardized environment [8,9]. Some programs have implemented mock OSCEs to better prepare their residents, and these practice sessions have proven to be highly valuable in enhancing skills and confidence [9].

Despite the importance of POCUS in anesthesiology, few published studies have examined the use of a POCUS OSCE to assess the competency of graduating anesthesiology residents [9]. Moreover, existing studies have not utilized independent evaluators to assess the skills of the testing residents. To address these gaps, we conducted a study to assess the competency of graduating anesthesiology residents from two accredited training programs using an indication, acquisition, interpretation, and medical decision-making (I-AIM)-based POCUS OSCE. In our study design, faculty from one program assessed the residents from the other program and vice versa, ensuring an independent, unbiased evaluation.

The primary outcome of our study was the POCUS OSCE scores of the graduating residents. The secondary outcome focused on resident feedback regarding their POCUS education. Our objective was to evaluate the competency of the graduating residents and identify the strengths and weaknesses of each program's POCUS training. The insights gained from this study could have broader implications for other residency programs, potentially leading to improvements in POCUS education and producing more proficient clinicians.

## Materials and methods

Ethical approval

All experimental protocols were exempt from the Institutional Review Boards (IRBs) at the University of Iowa and were deemed not human research at the University of Minnesota. Our study adhered to the ethical standards set forth by the Declaration of Helsinki, except for registration in a database. Due to the nature of the study, written informed consent was exempted for all participants prior to enrollment, as per the IRBs of both institutions. Verbal consent was obtained from all participants.

Study population and enrollment

Participants for this cross-sectional observational study were recruited from the directories of the University of Iowa and the University of Minnesota. A preapproved email invitation was sent to fourth-year anesthesiology resident physicians in the Department of Anesthesia at the University of Iowa and the University of Minnesota. The study population consisted exclusively of licensed anesthesia graduating residents from the final year of an anesthesiology residency. We excluded certified registered nurse anesthetists (CRNAs), anesthesiologist assistants (AAs), and medical students to maintain a homogeneous study group. No additional inclusion or exclusion criteria were applied. The study was done between 5/01/2022 and 6/30/2023.

Study protocol

The selection of principal investigators (PIs) at each site was based on their extensive experience with POCUS education. Each PI was required to be an institutional champion of resident POCUS education, fellowship-trained in cardiac anesthesiology and/or critical care medicine, and certified by the American Society of Anesthesiologists (ASA) POCUS [10] or another nationally recognized society for POCUS-related educational activities. The research team utilized the recently updated ACGME Anesthesiology Core Competencies and the relevant supplemental guide to prepare the mock exam assessments [11]. Additionally, the team referenced the ABA APPLIED exam content outline to determine the overall examination structure. At the time of the first mock assessment for the applied ABA, candidates were expected to demonstrate competency in the POCUS cardiac module only. However, the research team included both POCUS cardiac and lung ultrasound assessments to prepare for future ABA expectations. Prior to entering the examination room, each resident was required to complete a pre-assessment survey.

To assess the residents, various combinations of text forms were created, each evaluating one POCUS cardiac and one lung ultrasound view. Each resident was assessed by independent examiners from outside the Department of Anesthesia at each center. The University of Iowa had more residents, and hence two examiners were utilized, whereas only one examiner evaluated at the University of Minnesota. For the residents at the University of Iowa, the assessments were conducted by a physician from emergency medicine, certified in POCUS, and faculty from the University of Minnesota. Similarly, the assessments of residents at the University of Minnesota were conducted by anesthesiology faculty with POCUS expertise from the University of Iowa.

During the assessment, each examiner instructed the resident to perform one POCUS cardiac view and one lung ultrasound view. Residents were evaluated on their ability to position the patient appropriately, select the correct probe (especially for lung ultrasound), identify relevant anatomical structures, and respond to interpretive questions. The test forms included the parasternal long axis (PSL), parasternal short axis (PSS), apical four-chamber (A4), and subcostal views for POCUS cardiac assessments. For lung ultrasound views, the forms included assessments for pneumothorax (PN) and pleural effusion (PL) based on the Blue protocol and the Focused-Assessed Transthoracic Echocardiography (FATE) protocol [12, 13]. The timing of the assessments was aligned with the ABA APPLIED exam, providing each resident a total of three minutes from the moment they placed the probe on the simulated patient to complete the required examination and answer the relevant questions.

OSCE structure and timing

Station 1 (Knowledge)

This included 10 multiple-choice questions (MCQs; cardiopulmonary POCUS indications and principles); score = number correct (0-10) (Appendix A).

Station 2 (Interpretation/Management)

This included three vignettes from sets A/B/C (e.g., tamponade physiology, right ventricular strain, and pneumothorax/pleural effusion patterns). Each vignette required: (a) key sonographic findings, (b) synthesis/diagnosis, and (c) initial management step. Scoring per item (correct/incorrect) (Appendix B).

Station 3 (Acquisition on Live Models)

One cardiac view (PLAX, PSAX, A4C, or subcostal) and one lung view (pneumothorax or pleural effusion). Time limit: three minutes per view from probe contact. Assessed: patient positioning, probe selection, window optimization, anatomic landmarking, and saved image.

Rubric

Dichotomous/trichotomous items (Yes=1; Somewhat=0.5; No=0) summed to a total view score; the examiner also recorded a global 1-5 competency anchor for each view.

Station 4 (Post-assessment Survey)

Ten Likert items (1-5) on perceived preparedness, realism, feedback utility, and curricular suggestions (Appendix C).

Performance evaluation using rubric scoring

Each POCUS view was scored in a dichotomous (Yes/No) or trichotomous (Yes/Somewhat/No) manner. A score of yes = 1 point, somewhat = 0.5 points, and no = 0 points. In addition to scoring for each component, the examiner also recorded the time required for the acquisition of each view and the professionalism during the exam. The overall POCUS competency score was scored on a five-point Likert scale. Professionalism was evaluated based on the resident's interactions with the simulated patient and examiner [9].

Data collection

Each PI was responsible for entering all required data, including rubric scores for simulated patient assessments, knowledge test scores, scores from the pathology sections, and post-survey responses. The collected data were transmitted to the lead institution, the University of Minnesota, for analysis under a common data share agreement approved by the IRBs. This centralized data collection process ensured consistency and reliability in the analysis of the study results.

Data analysis

Measures are summarized as frequency (percentage) and mean (standard deviation) for categorical and continuous variables. Comparisons between sites (Minnesota and Iowa) are summarized using the median (first quartile, third quartile) with a Wilcoxon rank sum test to compare groups for continuous outcomes to account for the lack of normality in the outcomes. Fisher’s exact test is used for categorical comparisons to account for small, expected cell counts. P-values less than 0.05 were considered statistically significant. All analyses are completed with R v4.1.0 (The R Core Team, R Foundation for Statistical Computing, Vienna, Austria).

## Results

Over a two-year period, a total of 24 residents at the University of Iowa (13 in 2022 and 11 in 2023) and 12 students at the University of Minnesota (six in 2022 and six in 2023) were included in the study. Descriptive summaries of training outcomes and scenarios are presented in Table 1 for baseline knowledge, Table 2 for image-related outcomes, Table 3 for pathology-related outcomes, and Table 4 for post-survey questions. Since students only reviewed one of three pathology-related scenarios (A, B, or C), Table 3 also includes a composite measure for whether the first, second, or third pathology-related skill was met.

**Table 1:** Baseline knowledge: related descriptive summaries Values expressed are N(%). Q: Question

Covariate	Overall	University of Iowa	University of Minnesota
	(N=40)	(N=24)	(N=12)
Q1	23 (63.9%)	15 (62.5%)	8 (66.7%)
Q2	29 (80.6%)	21 (87.5%)	8 (66.7%)
Q3	25 (69.4%)	13 (54.2%)	12 (100.0%)
Q4	17 (47.2%)	10 (41.7%)	7 (58.3%)
Q5	15 (41.7%)	10 (41.7%)	5 (41.7%)
Q6	28 (77.8%)	20 (83.3%)	8 (66.7%)
Q7	34 (94.4%)	23 (95.8%)	11 (91.7%)
Q8	32 (88.9%)	22 (91.7%)	10 (83.3%)
Q9	27 (75.0%)	18 (75.0%)	9 (75.0%)
Q10	28 (80.0%)	18 (78.3%)	10 (83.3%)
Missing	1	1	0

**Table 2:** Image-related descriptive summaries Values are expressed are mean (SD) or N(%). A4: apical four-chamber; S4: subcostal four-chamber; PSL: parasternal long axis; PSS: parasternal short axis; PL: pleural; PN: pneumothorax

Covariate	Overall	University of Iowa	University of Minnesota
	(N=40)	(N=24)	(N=16)
A4 professionalism	17 (100.0%)	13 (100.0%)	4 (100.0%)
Missing	19	11	8
S4 professionalism	17 (100.0%)	11 (100.0%)	6 (100.0%)
Missing	19	13	6
PSL professionalism	20 (100.0%)	13 (100.0%)	7 (100.0%)
Missing	16	11	5
PSS professionalism	18 (100.0%)	11 (100.0%)	7 (100.0%)
Missing	18	13	5
PL professionalism	34 (97.1%)	24 (100.0%)	10 (90.9%)
Missing	1	0	1
PN Professionalism	34 (100.0%)	24 (100.0%)	10 (100.0%)
Missing	2	0	2
A4 total score	3.35 (1.41)	3.54 (1.13)	2.75 (2.22)
Missing	19 (52.8%)	11 (45.8%)	8 (66.7%)
S4 total score	3.12 (1.5)	2.45 (1.21)	4.33 (1.21)
Missing	19 (52.8%)	13 (54.2%)	6 (50.0%)
PSL total score	4.4 (1.05)	4.23 (1.17)	4.71 (0.76)
Missing	16 (44.4%)	11 (45.8%)	5 (41.7%)
PSS total score	4.83 (0.71)	4.73 (0.9)	5.0 (0.0)
Missing	18 (50.0%)	13 (54.2%)	5 (41.7%)
PL total score	3.11 (1.53)	2.78 (1.24)	3.75 (1.86)
Missing	1 (2.78%)	1 (4.17%)	0 (0.0%)
PN total score	4.36 (0.8)	4.38 (0.88)	4.32 (0.64)
Missing	1 (2.78%)	0 (0.0%)	1 (8.33%)
A4 competency	3.59 (1.23)	3.85 (0.99)	2.75 (1.71)
Missing	19 (52.8%)	11 (45.8%)	8 (66.7%)
S4 competency	3.35 (1.32)	2.82 (1.08)	4.33 (1.21)
Missing	19 (52.8%)	13 (54.2%)	6 (50.0%)
PSL competency	4.15 (1.14)	3.85 (1.21)	4.71 (0.76)
Missing	16 (44.4%)	11 (45.8%)	5 (41.7%)
PSS competency	4.44 (0.78)	4.09 (0.83)	5.0 (0.0)
Missing	18 (50.0%)	13 (54.2%)	5 (41.7%)
PL competency	3.11 (1.47)	2.58 (1.14)	4.17 (1.53)
PN competency	1 (2.78%)	0 (0.0%)	1 (8.33%)
Missing	3.35 (1.41)	3.54 (1.13)	2.75 (2.22)

**Table 3:** Pathology-related descriptive summaries Values expressed are N(%). A, B, and C are pathology/management question sets. Each set had three questions.

Covariate	Overall	University of Iowa	University of Minnesota
	(N=40)	(N=24)	(N=16)
A1	9 (90.0%)	6 (100.0%)	3 (75.0%)
Missing	26	18	8
A2	9 (90.0%)	5 (83.3%)	4 (100.0%)
Missing	26	18	8
A3	6 (60.0%)	3 (50.0%)	3 (75.0%)
Missing	26	18	8
B1	11 (91.7%)	7 (87.5%)	4 (100.0%)
Missing	24	16	8
B2	12 (100.0%)	8 (100.0%)	4 (100.0%)
Missing	24	16	8
B3	11 (91.7%)	8 (100.0%)	3 (75.0%)
Missing	24	16	8
C1	12 (85.7%)	9 (90.0%)	3 (75.0%)
Missing	22	14	8
C2	13 (92.9%)	10 (100.0%)	3 (75.0%)
Missing	22	14	8
C3	14 (100.0%)	10 (100.0%)	4 (100.0%)
Missing	22	14	8
ABC 1	32 (88.9%)	22 (91.7%)	10 (83.3%)
ABC 2	31 (86.1%)	21 (87.5%)	10 (83.3%)
ABC 3	26	18	8

**Table 4:** Post survey related descriptive summaries Values expressed are N(%). Q: question

Covariate	Overall	University of Iowa	University of Minnesota
	(N=40)	(N=24)	(N=16)
Post-survey Q1	4.75 (0.44)	4.75 (0.44)	4.75 (0.45)
Post-survey Q2	4.61 (0.6)	4.58 (0.65)	4.67 (0.49)
Post-survey Q3	4.5 (0.81)	4.58 (0.72)	4.33 (0.98)
Post-survey Q4	4.72 (0.45)	4.71 (0.46)	4.75 (0.45)
Post-survey Q5	4.69 (0.52)	4.58 (0.58)	4.92 (0.29)
Post-survey Q6	4.75 (0.55)	4.71 (0.55)	4.83 (0.58)
Post-survey Q7	4.83 (0.38)	4.79 (0.41)	4.92 (0.29)
Post-survey Q8	4.67 (0.53)	4.58 (0.58)	4.83 (0.39)
Post-survey Q9	4.72 (0.51)	4.67 (0.56)	4.83 (0.39)
Post-survey Q10	4.75 (0.44)	4.67 (0.48)	4.92 (0.29)

For outcomes measured on a five-point scale, there were significantly higher scores for the Univesrity of Minnesota relative to the University of Iowa for the S4 total score (p=0.019; median 5 versus 2), S4 competency (p=0.033; median 5 versus 3), PSS competency (p=0.005; median 5 versus 4), PL competency (p=0.005; median 5 versus 2.5), and PN competency (p=0.021; 5 versus 4). All other total scores, competencies, and post-survey questions were not significantly different (p>0.05; Table 5).

**Table 5:** Comparison of continuous outcomes between sites between the University of Iowa and University of Minnesota Summary statistics are presented as median (Q1, Q3) with p-value from the nonparametric Wilcoxon rank-sum test. Q: question; A4: apical four-chamber; S4: subcostal four-chamber; PSL: parasternal long axis; PSS: parasternal short axis; PN: pneumothorax; PL: pleural effusion

Outcome	University of Iowa Median (Q1, Q3) (N)	University of Minnesota Median (Q1, Q3) (N)	p-value
A4 total score	4 (3, 4) (N=13)	3 (1.5, 4.25) (N=4)	0.637
S4 total score	2 (2, 3) (N=11)	5 (4.25, 5) (N=6)	0.019
PSL total score	5 (4, 5) (N=13)	5 (5, 5) (N=7)	0.242
PSS total score	5 (5, 5) (N=11)	5 (5, 5) (N=7)	0.494
PL total score	3 (2, 3.5) (N=23)	5 (1.75, 5) (N=12)	0.107
PN total score	5 (3.75, 5) (N=24)	4 (4, 5) (N=11)	0.53
A4 competency	4 (3, 5) (N=13)	2.5 (1.75, 3.5) (N=4)	0.198
S4 competency	3 (2, 3) (N=11)	5 (4.25, 5) (N=6)	0.033
PSL competency	4 (3, 5) (N=13)	5 (5, 5) (N=7)	0.079
PSS competency	4 (4, 4.5) (N=11)	5 (5, 5) (N=7)	0.005
PL competency	2.5 (2, 3) (N=24)	5 (4.25, 5) (N=12)	0.005
PN competency	4 (3, 4) (N=24)	5 (4, 5) (N=11)	0.021
Post-Survey Q1	5 (4.75, 5) (N=24)	5 (4.75, 5) (N=12)	1
Post-survey Q2	5 (4, 5) (N=24)	5 (4, 5) (N=12)	0.887
Post-survey Q3	5 (4, 5) (N=24)	5 (4, 5) (N=12)	0.514
Post-survey Q4	5 (4, 5) (N=24)	5 (4.75, 5) (N=12)	0.812
Post-survey Q5	5 (4, 5) (N=24)	5 (5, 5) (N=12)	0.071
Post-survey Q6	5 (4.75, 5) (N=24)	5 (5, 5) (N=12)	0.306
Post-survey Q7	5 (5, 5) (N=24)	5 (5, 5) (N=12)	0.363
Post-survey Q8	5 (4, 5) (N=24)	5 (5, 5) (N=12)	0.202
Post-survey Q9	5 (4, 5) (N=24)	5 (5, 5) (N=12)	0.41
Post-survey Q10	5 (4, 5) (N=24)	5 (5, 5) (N=12)	0.112

There were no significant differences in total scores, competencies, or post-survey questions between 2022 and 2023 for either the University of Minnesota (Table 6; p>0.05) or the University of Iowa (Table 7; p>0.05). 

**Table 6:** Comparison of continuous outcomes between sites within the University of Minnesota for 2022 and 2023 Summary statistics are presented as median (Q1, Q3) with p-value from the nonparametric Wilcoxon rank-sum test. Q: question; A4: apical four-chamber; S4: subcostal four-chamber; PSL: parasternal long axis; PSS: parasternal short axis; PL: pleural effusion; PN: pneumothorax

Outcome	University of Minnesota 2022 Median (Q1, Q3) (N)	University of Minnesota 2023 Median (Q1, Q3) (N)	p-value
A4 total score	2 (1, 3) (N=3)	5 (5, 5) (N=1)	0.5
S4 total score	4 (3, 4.5) (N=3)	5 (5, 5) (N=3)	0.197
PSL total score	5 (4, 5) (N=3)	5 (5, 5) (N=4)	0.386
PSS total score	5 (5, 5) (N=3)	5 (5, 5) (N=4)	-
PL total score	3.5 (1.25, 5) (N=6)	5 (5, 5) (N=6)	0.336
PN total score	4 (4, 4) (N=5)	4.75 (4.125, 5) (N=6)	0.169
A4 competency	2 (1.5, 2.5) (N=3)	5 (5, 5) (N=1)	0.5
S4 competency	4 (3, 4.5) (N=3)	5 (5, 5) (N=3)	0.197
PSL competency	5 (4, 5) (N=3)	5 (5, 5) (N=4)	0.386
PSS competency	5 (5, 5) (N=3)	5 (5, 5) (N=4)	-
PL competency	5 (2.75, 5) (N=6)	5 (5, 5) (N=6)	0.527
PN competency	4 (4, 4) (N=5)	5 (5, 5) (N=6)	0.052
Post-survey Q1	5 (4.25, 5) (N=6)	5 (5, 5) (N=6)	0.595
Post-survey Q2	5 (4.25, 5) (N=6)	5 (4.25, 5) (N=6)	1
Post-survey Q3	5 (4.25, 5) (N=6)	4.5 (4, 5) (N=6)	0.652
Post-survey Q4	5 (4.25, 5) (N=6)	5 (5, 5) (N=6)	0.595
Post-survey Q5	5 (5, 5) (N=6)	5 (5, 5) (N=6)	0.405
Post-survey Q6	5 (5, 5) (N=6)	5 (5, 5) (N=6)	0.405
Post-survey Q7	5 (5, 5) (N=6)	5 (5, 5) (N=6)	0.405
Post-survey Q8	5 (4.25, 5) (N=6)	5 (5, 5) (N=6)	0.174
Post-survey Q9	5 (4.25, 5) (N=6)	5 (5, 5) (N=6)	0.174
Post-survey Q10	5 (5, 5) (N=6)	5 (5, 5) (N=6)	0.405

**Table 7:** Comparison of continuous outcomes between sites for University of Iowa for 2022 and 2023 Summary statistics presented as median (Q1, Q3) with p-value from the nonparametric Wilcoxon rank-sum test. Q: question; A4: apical four-chamber; S4: subcostal four-chamber; PSL: parasternal long axis; PSS: parasternal short axis; PL: pleural effusion; PN: pneumothorax

Outcome	University of Iowa 2022 Median (Q1, Q3) (N)	University of Iowa 2023 Median (Q1, Q3) (N)	p-value
A4 total score	3 (3, 4) (N=7)	4 (4, 4) (N=6)	0.449
S4 total score	2 (2, 3.5) (N=6)	2 (1, 3) (N=5)	0.386
PSL total score	4 (4, 5) (N=7)	5 (4.25, 5) (N=6)	0.636
PSS total score	5 (5, 5) (N=6)	5 (5, 5) (N=5)	0.361
PL total score	2 (2, 3) (N=12)	3 (2, 4) (N=11)	0.265
PN total score	5 (3, 5) (N=13)	5 (4.5, 5) (N=11)	0.381
A4 competency	3 (3, 4) (N=7)	4.5 (4, 5) (N=6)	0.117
S4 competency	2 (2, 2.75) (N=6)	3 (3, 3) (N=5)	0.147
PSL competency	4 (3, 4.5) (N=7)	4.5 (3.25, 5) (N=6)	0.764
PSS competency	4 (4, 4.75) (N=6)	4 (4, 4) (N=5)	0.454
PL competency	2 (2, 3) (N=13)	3 (2, 3.5) (N=11)	0.319
PN competency	4 (3, 4) (N=13)	4 (3, 4) (N=11)	0.856
Post-survey Q1	5 (5, 5) (N=13)	5 (4.5, 5) (N=11)	0.847
Post-survey Q2	5 (4, 5) (N=13)	5 (4, 5) (N=11)	0.601
Post-survey Q3	5 (4, 5) (N=13)	5 (4.5, 5) (N=11)	0.527
Post-survey Q4	5 (4, 5) (N=13)	5 (4.5, 5) (N=11)	0.883
Post-survey Q5	5 (5, 5) (N=13)	4 (4, 5) (N=11)	0.109
Post-survey Q6	5 (5, 5) (N=13)	5 (4.5, 5) (N=11)	0.759
Post-survey Q7	5 (4, 5) (N=13)	5 (5, 5) (N=11)	0.217
Post-survey Q8	5 (4, 5) (N=13)	5 (4, 5) (N=11)	0.973
Post-survey Q9	5 (4, 5) (N=13)	5 (4.5, 5) (N=11)	1
Post-survey Q10	5 (4, 5) (N=13)	5 (4, 5) (N=11)	0.804

For binary outcomes, the only significant difference between the University of Iowa and the University of Minnesota was with respect to question 3 (54.2% (13/24); University of Iowa versus University of Minnesota: 100% (12/12)). All other questions with a binary outcome, evaluations of professionalism, and scenario-specific scores were not statistically different (p>0.05 for all; Table 8).

**Table 8:** Comparison of categorical outcomes between University of Iowa and University of Minnesota sites with p-values from the Fisher’s exact test Q: question; A, B, C: pathology question sets each with three questions; A4: apical four-chamber; S4: subcostal four-chamber; PSL: parasternal long axis; PSS: parasternal short axis; PL: pleural effusion; PN: pneumothorax

Outcome	University of Iowa % (x/N)	University of Minnesota % (x/N)	p-value
Q1	62.5% (15/24)	66.7% (8/12)	1.000
Q2	87.5% (21/24)	66.7% (8/12)	0.190
Q3	54.2% (13/24)	100.0% (12/12)	0.006
Q4	41.7% (10/24)	58.3% (7/12)	0.483
Q5	41.7% (10/24)	41.7% (5/12)	1.000
Q6	83.3% (20/24)	66.7% (8/12)	0.397
Q7	95.8% (23/24)	91.7% (11/12)	1.000
Q8	91.7% (22/24)	83.3% (10/12)	0.588
Q9	75.0% (18/24)	75.0% (9/12)	1.000
Q10	78.3% (18/23)	83.3% (10/12)	1.000
A4 Professionalism	100.0% (13/13)	100.0% (4/4)	1.000
S4 professionalism	100.0% (11/11)	100.0% (6/6)	1.000
PSL professionalism	100.0% (13/13)	100.0% (7/7)	1.000
PSS professionalism	100.0% (11/11)	100.0% (7/7)	1.000
PL professionalism	100.0% (24/24)	90.9% (10/11)	0.314
PN professionalism	100.0% (24/24)	100.0% (10/10)	1.000
A1	100.0% (6/6)	75.0% (3/4)	0.400
A2	83.3% (5/6)	100.0% (4/4)	1.000
A3	50.0% (3/6)	75.0% (3/4)	0.571
B1	87.5% (7/8)	100.0% (4/4)	1.000
B2	100.0% (8/8)	100.0% (4/4)	1.000
B3	100.0% (8/8)	75.0% (3/4)	0.333
C1	90.0% (9/10)	75.0% (3/4)	0.505
C2	100.0% (10/10)	75.0% (3/4)	0.286
C3	100.0% (10/10)	100.0% (4/4)	1.000
ABC1	91.7% (22/24)	83.3% (10/12)	0.588
ABC2	95.8% (23/24)	91.7% (11/12)	1.000
ABC3	87.5% (21/24)	83.3% (10/12)	1.000

There were no significant differences between the 2022 and 2023 cohorts within the University of Minnesota (p>0.05 for all; Table 9) or within the University of Iowa (p>0.05 for all; Table 10). 

**Table 9:** Comparison of categorical outcomes within University of Minnesota between 2022 and 2023, with p-values calculated using the Fisher’s exact test. Q: question; A4: apical four-chamber; S4: subcostal four-chamber; PSS: parasternal short axis; PSL: parasternal long axis; PL: pleural; PN: pneumothorax; A, B, C: pathology question sets with three questions in each set

Outcome	University of Minnesota 2022 % (x/N)	University of Minnesota 2023 % (x/N)	p-value
Q1	66.7% (4/6)	66.7% (4/6)	1.000
Q2	66.7% (4/6)	66.7% (4/6)	1.000
Q3	100.0% (6/6)	100.0% (6/6)	1.000
Q4	66.7% (4/6)	50.0% (3/6)	1.000
Q5	16.7% (1/6)	66.7% (4/6)	0.242
Q6	33.3% (2/6)	100.0% (6/6)	0.061
Q7	83.3% (5/6)	100.0% (6/6)	1.000
Q8	83.3% (5/6)	83.3% (5/6)	1.000
Q9	66.7% (4/6)	83.3% (5/6)	1.000
Q10	66.7% (4/6)	100.0% (6/6)	0.455
A4 professionalism	100.0% (3/3)	100.0% (1/1)	1.000
S4 professionalism	100.0% (3/3)	100.0% (3/3)	1.000
PSL professionalism	100.0% (3/3)	100.0% (4/4)	1.000
PSS professionalism	100.0% (3/3)	100.0% (4/4)	1.000
PL professionalism	83.3% (5/6)	100.0% (5/5)	1.000
PN professionalism	100.0% (4/4)	100.0% (6/6)	1.000
A1	100.0% (2/2)	50.0% (1/2)	1.000
A2	100.0% (2/2)	100.0% (2/2)	1.000
A3	100.0% (2/2)	50.0% (1/2)	1.000
B1	100.0% (2/2)	100.0% (2/2)	1.000
B2	100.0% (2/2)	100.0% (2/2)	1.000
B3	50.0% (1/2)	100.0% (2/2)	1.000
C1	50.0% (1/2)	100.0% (2/2)	1.000
C2	50.0% (1/2)	100.0% (2/2)	1.000
C3	100.0% (2/2)	100.0% (2/2)	1.000
ABC1	83.3% (5/6)	83.3% (5/6)	1.000
ABC2	83.3% (5/6)	100.0% (6/6)	1.000
ABC3	83.3% (5/6)	83.3% (5/6)	1.000

**Table 10:** Comparison of categorical outcomes within University of Iowa between 2022 and 2023, with p-values calculated using the Fisher’s exact test. Q: question; A4: apical four-chamber; S4: subcostal four-chamber; PSS: parasternal short axis; PSL: parasternal long axis; PL: pleural; PN: pneumothorax; A, B, C: pathology question sets with three questions in each set

Outcome	University of Iowa 2022 % (x/N)	University of Iowa 2023 % (x/N)	p-value
Q1	76.9% (10/13)	45.5% (5/11)	0.206
Q2	76.9% (10/13)	100.0% (11/11)	0.223
Q3	53.8% (7/13)	54.5% (6/11)	1.000
Q4	30.8% (4/13)	54.5% (6/11)	0.408
Q5	23.1% (3/13)	63.6% (7/11)	0.095
Q6	76.9% (10/13)	90.9% (10/11)	0.596
Q7	92.3% (12/13)	100.0% (11/11)	1.000
Q8	92.3% (12/13)	90.9% (10/11)	1.000
Q9	76.9% (10/13)	72.7% (8/11)	1.000
Q10	69.2% (9/13)	90.0% (9/10)	0.339
A4 professionalism	100.0% (7/7)	100.0% (6/6)	1.000
S4 professionalism	100.0% (6/6)	100.0% (5/5)	1.000
PSL professionalism	100.0% (7/7)	100.0% (6/6)	1.000
PSS professionalism	100.0% (6/6)	100.0% (5/5)	1.000
PL Professionalism	100.0% (13/13)	100.0% (11/11)	1.000
PN professionalism	100.0% (13/13)	100.0% (11/11)	1.000
A1	100.0% (4/4)	100.0% (2/2)	1.000
A2	75.0% (3/4)	100.0% (2/2)	1.000
A3	50.0% (2/4)	50.0% (1/2)	1.000
B1	75.0% (3/4)	100.0% (4/4)	1.000
B2	100.0% (4/4)	100.0% (4/4)	1.000
B3	100.0% (4/4)	100.0% (4/4)	1.000
C1	100.0% (5/5)	80.0% (4/5)	1.000
C2	100.0% (5/5)	100.0% (5/5)	1.000
C3	100.0% (5/5)	100.0% (5/5)	1.000
ABC1	92.3% (12/13)	90.9% (10/11)	1.000
ABC2	92.3% (12/13)	100.0% (11/11)	1.000
ABC3	84.6% (11/13)	90.9% (10/11)	1.000

## Discussion

POCUS training has been included as a core competency by the ACGME [3], and POCUS testing has been included in the OSCE in the ABA since 2022 [4].

Assessing the effectiveness of POCUS training in residents is undoubtedly important, but only a few studies have assessed training efficiency. Davinder Ramsingh et al. have published the impact of having a structured curriculum on improving imaging capability, recognizing pathology, and satisfaction [14]. Sanders et al. have used a pretest and posttest questionnaire by faculty from the same department to evaluate the efficiency of a structured POCUS teaching curriculum. They did not, however, evaluate the ability of the residents to acquire or interpret images [5]. Some others used a simulator to train residents how to obtain and interpret images and manage cardiac arrest patients, and the efficacy of training was measured by pre- and post-tests [15].

OSCEs have long been shown to be an effective method of assessing competency [8]. More recent studies have reported the usefulness of POCUS OSCEs in preparing residents for their board exams [9]. To our knowledge, we are the first to evaluate the multiple aspects of POCUS training in graduating residents using the OSCE format and using external examiners.

The use of external evaluators ensured an unbiased assessment. Faculty from one institution evaluated residents from the other, eliminating potential biases associated with internal assessments. Additionally, the OSCE format allowed for a comprehensive evaluation of the I-AIM framework, covering the essential components of POCUS competency. This methodology aligns with best practices for assessing procedural skills and provides valuable feedback for both residents and training programs.

We conducted a study on graduating anesthesia residents from two major residency programs: the University of Iowa and the University of Minnesota. We selected the graduating class of residents, as they represent the most highly trained individuals prior to entering independent practice.

Previous studies have evaluated training efficiency by assessing various aspects, such as knowledge and the ability to obtain and interpret images [6]. However, achieving success in POCUS in clinical practice requires familiarity with equipment and competency across all components of the I-AIM model [16]. The I-AIM model encompasses four essential tenets: knowledge, image acquisition, image interpretation, and management of patients using information derived from interpreted images. By modeling our study on the I-AIM framework, we were able to comprehensively assess the residents’ POCUS skills, ensuring all critical components are evaluated for clinical readiness.

The four stations in our study tested different aspects of resident training. Station 1 tested the knowledge and, to some extent, patient management components based on 10 MCQs. MCQs have been shown to be an effective way of testing knowledge [17].

Station 2 tested the image interpretation and patient management portions of learning based on slides depicting pathology, and Station 3 tested the ability of the residents to acquire images on models. Station 4 was the post-survey questionnaire to assess resident satisfaction.

We chose to use live models instead of a simulator to test professionalism and the ability of the residents to optimize the POCUS image by appropriate positioning.

The residents at the University of Minnesota did better than their University of Iowa counterparts when it came to image acquisition. There was no statistical difference in the knowledge portion (MCQs), the image interpretation, or the post-survey portions of the study.

A more detailed look at the POCUS curriculum might explain some of the observed differences. The ultrasound curricula at the two institutions, though similar in concept, do have some key differences. At the University of Iowa, the training starts during the internship with a 10-hour combined comprehensive lecture and hands-on workshop. While there is no structured ultrasound POCUS rotation, POCUS education is integrated into the transesophageal echocardiography (TEE) rotation and reinforced during clinical scenarios. Participation in the ASA POCUS certificate course is optional but encouraged. The University of Iowa had a POCUS and TEE simulator that was easily available to the residents for POCUS and TEE training.

At the University of Minnesota, the POCUS training starts during the Clinical Anesthesiology Year 1 (CA-1) of the residency with a 10-hour FATE workshop for POCUS transthoracic and pleural exam assessment. It is followed by a workshop on pig heart dissection to help residents understand the correlation between cardiac anatomy and echocardiography. Between December and March, there is a series of TEE workshops on a simulator. A series of workshops is conducted between January and May of every year on gastric, airway, lung, and FAST POCUS entities. All the workshops require e-learning prior to the workshop, and the POCUS skills learned in the prior workshops are reinforced in subsequent workshops. Except for the FATE course, every resident goes through a series of workshops every year. Apart from the series of workshops, there is a dedicated ultrasound rotation and curriculum, where the residents are required to record a minimum of 50 supervised FATE exams, 50 gastric exams, 50 lung ultrasound exams, 20 airway exams, and 20 FAST exams.

At the University of Minnesota, self-motivated residents were given additional training to become instructors at POCUS sessions. Such near-peer trainers have been used effectively with medical students before [18]. This additional training may have helped the residents perform better than their Iowa counterparts.

A common theme between both programs was repetition in POCUS skills. It has been established that repetition in POCUS skills is important, and the plateau for learning was achieved at 16 exams for lung POCUS and 25 exams for cardiac POCUS [19]. The residents in both programs had more scans than the minimum number of scans to achieve a plateau in competency. One of the models at the University of Iowa had difficult subcostal echocardiographic windows even for the experienced examiners. It's known that some body habitus types can make echocardiographic windows difficult [20]. The differences in the training curriculum and body habitus of the models can explain why residents at the University of Minnesota performed better than their Iowa counterparts in image acquisition.

To our knowledge, we might be the first to study the effectiveness of POCUS teaching objectively using an external examiner and OSCE. A structured curriculum with clear expectations and reinforcing skills with repeated scanning under supervision will enhance resident POCUS learning. Using an unbiased external examiner to assess the different aspects of learning may be useful in providing objective feedback to the residents and in identifying areas for improvement. In our study, we included only cardiac and lung ultrasound components. As more POCUS areas are included in the ABA OSCE, objective evaluation of learned POCUS skills using an unbiased external examiner will be valuable in assessing training efficiency and providing feedback to trainees.

Limitations

Despite its strengths, our study has several limitations. First, the sample size was relatively small, with only two cohorts, one from each program, which may limit the generalizability of the findings. Second, we focused exclusively on cardiac and lung ultrasound assessments, whereas other POCUS applications, such as gastric and airway exams, were not evaluated. Having two examiners in the Iowa group versus one in the Minnesota group and a model with difficult sonographic windows may also account for some of the differences. We also had four residents not participate as planned, limiting our sample size. Expanding the scope of assessments in future studies could provide a more comprehensive understanding of POCUS training effectiveness.

## Conclusions

To our knowledge, we might be the first to study the effectiveness of POCUS teaching objectively using an external examiner and OSCE. A structured curriculum with clear expectations and repeated scanning under supervision will enhance resident POCUS learning. Using an unbiased external examiner to assess the different aspects of learning may be useful in providing objective feedback to the residents and in identifying areas for improvement. In our study, we included only cardiac and lung ultrasound components. As more POCUS areas are included in the ABA OSCE, objective evaluation of learned POCUS skills using an unbiased external examiner will be valuable in assessing training efficiency and providing feedback to trainees.

## Figures and Tables

**Figure 1:**
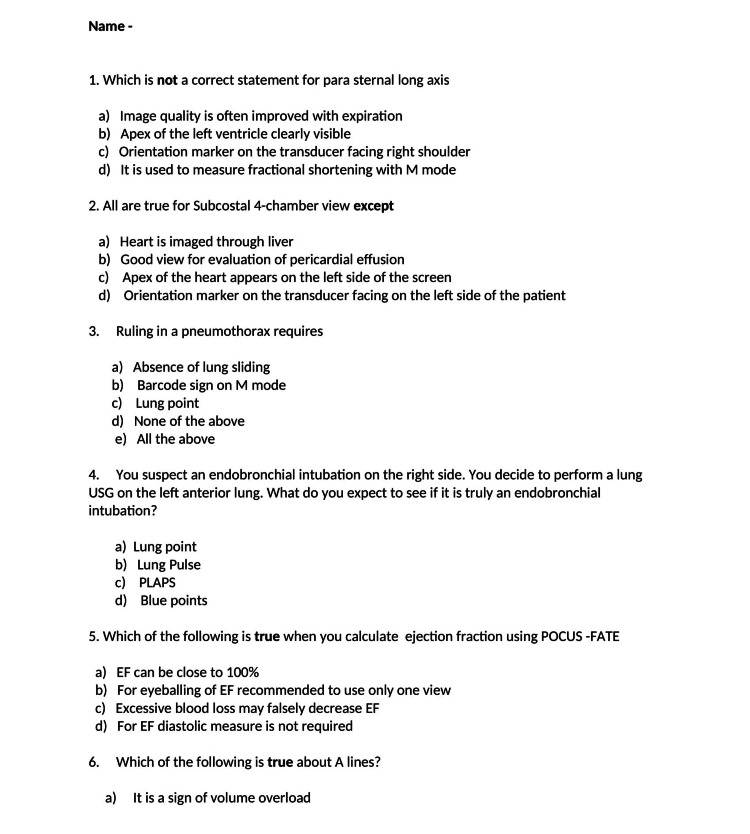
Station 1: Multiple-Choice Questions (Part 1)

**Figure 2:**
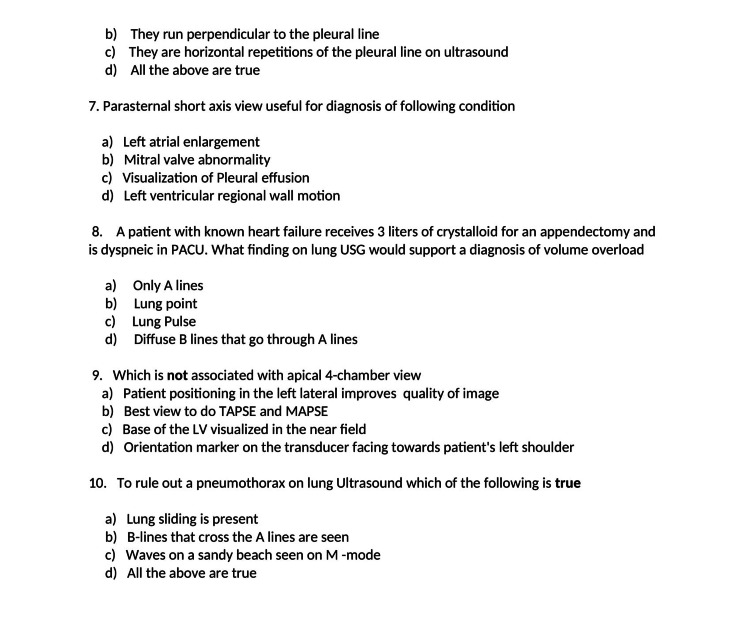
Station 1: Multiple-Choice Questions (Part 2)

**Video 1:** Station 2 (Interpretation/Management): Part A

**Video 2:** Station 2 (Interpretation/Management): Part B

**Video 3:** Station 2 (Interpretation/Management): Part C

**Figure 3:**
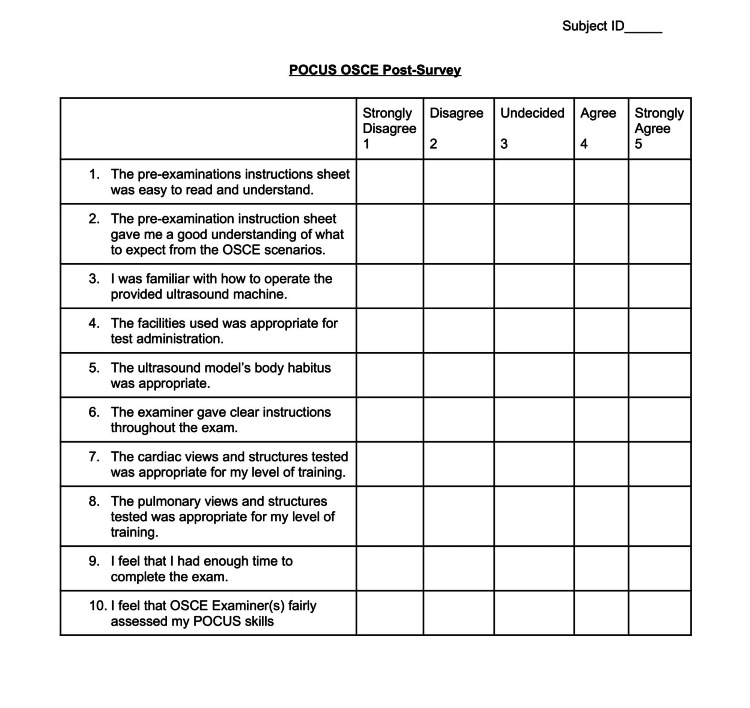
Station 4 (Post-assessment Survey) POCUS:  point-of-care ultrasound; OSCE: Objective Structured Clinical Examinations

## Appendices

Appendix A

Appendix B

Appendix C
